# Supplementary material for: Robotic versus laparoscopic total mesorectal excision with lateral lymph node dissection for advanced rectal cancer: A systematic review and meta-analysis
Source: PLoS One. 2024 May 29;19(5):e0304031. doi: 10.1371/journal.pone.0304031 (PMC11135705; doi:10.1371/journal.pone.0304031)
Supplement: Attachment — Submitted filename: review.pdf [file pone.0304031.s002.pdf]

This study is a meta-analysis conducted to investigate the advantages of robotic surgery over laparoscopic surgery in performing Lateral Pelvic Node Dissection (LPND). Appropriate studies were identified and analyzed. However, I would like to inquire about a few points:

1. The introduction states that while the benefits of postoperative outcomes are identified, the oncological safety has not been clarified. However, the abstract states that this study will clarify postoperative outcomes. If the objective is to compare short-term outcomes, the introduction in the text needs to be refined.

2. In the introduction, the citation for the following sentence needs to be corrected: "The overall risk of recurrence following this strategy increases from 10 (2) to 30% in cases of lateral node involvement (2)."

3. In the introduction, the citation for the following sentence needs to be replaced with a more appropriate one: "In the East, promising results have been demonstrated when LPN metastasis (LPNM) is treated with TME and Lateral Pelvic Node Dissection (LPND) (1)."

4. In the methods, the paragraph on eligibility criteria's studies needs clarification: "We included all randomized controlled trials (RCTs) and controlled clinical trials (CCTs) that compared robotic Total Mesorectal Excision (RTME) with Laparoscopic Pelvic Lymph Node Dissection (LPND), and laparoscopic Total Mesorectal Excision (LTME) with LPND." Please verify if the term 'laparoscopic pelvic lymph node dissection' is incorrectly written

instead of 'lateral pelvic lymph node dissection.'

5. To analyze differences in anastomotic leakage, it would be beneficial to add the stoma rate to the results.

6. In the results, it would be advisable to include lymphocele, which is an LPND-specific complication.

7. Regarding the third paragraph of the discussion: "Because LPND requires only lymphatic tissue dissection, the lateral pelvic vascular and nervous complexes should be preserved during skeletonization." I disagree with this statement. I believe that if LPN has to be removed in proximity to certain structures, some vessels and nerves may need to be sacrificed. I would like to hear your opinion on this.

8. The use of abbreviations needs to be systematically reviewed throughout the text. Especially since LPN is described in the fifth paragraph of the discussion, it should have been detailed earlier. And as previously mentioned, the abbreviation "Laparoscopic Pelvic Lymph Node Dissection (LPND)" is inappropriate. There is a need for a clear review and arrangement of the abbreviations used throughout the text.
