# Supplementary material for: Robotic versus laparoscopic total mesorectal excision with lateral lymph node dissection for advanced rectal cancer: A systematic review and meta-analysis
Source: PLoS One. 2024 May 29;19(5):e0304031. doi: 10.1371/journal.pone.0304031 (PMC11135705; doi:10.1371/journal.pone.0304031)
Supplement: Attachment — Submitted filename: Reviewers queries responses.docx [file pone.0304031.s003.docx]

**Reviewer queries responses**

**Editor**
Q1. Regarding postoperative complications, more detailed descriptions are required for definitions and grades.

R1. We have added that mortality and morbidity were considered when they occurred within 90 and 30 days after rectal resection, respectively.
Q2. Will this review focus on both short-term and long-term results? It would be better to make a clear description of these.

R2. We have removed the term “long-term” and we have used “oncological” results only to avoid this doubt.
Q3. There are no RCT or PS matching studies, and the review contains considerable selection bias. Considering this, the review must not mislead the readers.

R3. Thank you for this valuable remark. We have mentioned this detail in the limitation of the study, reported in the risk of bias assessment of the retained studies, and it was a reason to decrease the level of evidence of the different conclusions after the GRADE assessment in the summary of findings.

**Reviewers**

**Reviewer #1:** 
Q1. As described in discussion, the studies including in this analysis are all retrospective studies, which lead to selection bias.
R1. Thank you for this valuable remark. We have mentioned this detail in the limitation of the study, reported in the risk of bias assessment of the retained studies, and it was a reason to decrease the level of evidence of the different conclusions after the GRADE assessment in the summary of findings.
Q2. The BMI of the cases is low (mean 21-23), so it should be described that this study is based on a biased case.
R2. Yes absolutely. We have also remarked on this point. However, it is explained by the origin of these studies from Asian countries that were well known with their low BMI. We have added this information in the discussion section and we have highlighted its interest for an external validity of our findings. Thank you for this remark.

Q3. The definition of each complication is not stated. The grade of the complication should also be described.
R3. We have added a definition of the different complications. Unfortunately, we cannot add additional information regarding the grade because it was not reported in the retained studies.

Q4. There are many typographical errors.

R4. We have corrected the different errors. Thank you

**Reviewer #2:**

Q1. The introduction states that while the benefits of postoperative outcomes are identified, the oncological safety has not been clarified. However, the abstract states that this study will clarify postoperative outcomes. If the objective is to compare short-term outcomes, the introduction in the text needs to be refined.

R1. We have removed this sentence from the introduction to alleviate this issue.

Q2. In the introduction, the citation for the following sentence needs to be corrected: "The overall risk of recurrence following this strategy increases from 10 (2) to 30% in cases of lateral node involvement (2)."

R2. We have corrected the references.

Q3. In the introduction, the citation for the following sentence needs to be replaced with a more appropriate one: "In the East, promising results have been demonstrated when LPN metastasis (LPNM) is treated with TME and Lateral Pelvic Node Dissection (LPND) (1)."

R3. We have added a reference to this sentence.

Q4. In the methods, the paragraph on eligibility criteria's studies needs clarification: "We included all randomized controlled trials (RCTs) and controlled clinical trials (CCTs) that compared robotic Total Mesorectal Excision (RTME) with Laparoscopic Pelvic Lymph Node Dissection (LPND), and laparoscopic Total Mesorectal Excision (LTME) with LPND." Please verify if the term 'laparoscopic pelvic lymph node dissection' is incorrectly written instead of 'lateral pelvic lymph node dissection.'
R4. We have corrected it.
Q5. To analyze differences in anastomotic leakage, it would be beneficial to add the stoma rate to the results.

R5. Unfortunately, the stoma rate was not reported in the included studies.
Q6. In the results, it would be advisable to include lymphocele, which is an LPND-specific complication.

R6. This outcome was not reported in the different included studies.
Q7. Regarding the third paragraph of the discussion: "Because LPND requires only lymphatic tissue dissection, the lateral pelvic vascular and nervous complexes should be preserved during skeletonization." I disagree with this statement. I believe that if LPN has to be removed in proximity to certain structures, some vessels and nerves may need to be sacrificed. I would like to hear your opinion on this.
R7. Yes absolutely. In some cases, we found a huge adhesion of metastasis LN and we are obliged to scarify certain structures. We have added this information to this sentence.
Q8. The use of abbreviations needs to be systematically reviewed throughout the text. Especially since LPN is described in the fifth paragraph of the discussion, it should have been detailed earlier. And as previously mentioned, the abbreviation "Laparoscopic Pelvic Lymph Node Dissection (LPND)" is inappropriate. There is a need for a clear review and arrangement of the abbreviations used throughout the text.

Q9. We have reviewed all the abbreviations.

**Reviewer #3:** 

Q1. The introduction is rather wordy and covers many different concepts. This should be further summarised in shortened paragraphs to discuss the pertinent issues related to the topic at hand.
R1. We have shortened the introduction.

Q2. The major limitation of the study is the lack of mature data and paucity of randomized trials, prospective studies, or propensity score matched studies which can control for confounding and selection bias.
R2. We have added this information in the limitations of our study.

Q3. Several RCTs and meta-analyses already exist comparing Lap vs Robotic TME. The authors should decide if the study is to compare Lap TME + LPND vs RTME + LPND as currently is, or to focus on the Lap LPND vs Robotic LPND aspect – this is an important distinction as several important quality control and oncological parameters are missing should TME be included, such as rates of complete TME, distal resection margin, CRM positivity, and conversion rates. I suggest that the authors focus on the LPND aspect as this is relatively novel while there is an abundance of data on approaches to TME.
R3. Thank you for this remark. At the beginning of our study, we performed literature research for all the studies comparing robotic and laparoscopic LPND. We have found studies of urology, digestive disease, and gynaecology. The most relevant issue was that the study population of the PICO that we used was very heterogeneous and it was impossible to pool the outcomes with a huge heterogeneity among the different studies in the majority of the outcomes. We thought that a focus only LPND is an excellent subject of a systematic review and not a meta-analysis.

Q4. It should be strongly emphasized that the grade of evidence is low to very low across all measures of outcomes resulting in an inability to draw definite conclusions.

R4. Yes, absolutely we have mentioned this information.
