# Supplementary material for: Robotic versus laparoscopic total mesorectal excision with lateral lymph node dissection for advanced rectal cancer: A systematic review and meta-analysis
Source: PLoS One. 2024 May 29;19(5):e0304031. doi: 10.1371/journal.pone.0304031 (PMC11135705; doi:10.1371/journal.pone.0304031)
Supplement: Attachment — Submitted filename: Reviewers queries.docx [file pone.0304031.s004.docx]

**Reviewers’ queries responses**

Reviewer #2:

You've worked hard on the revisions. I think your excellent research results have been sufficiently supplemented. I would like to make one request. It seems necessary to review all the citations in the discussion. There are minor issues such as some references having incorrect numbers or duplications. I recommend reviewing them once again.

Response :

Thank you for the remarks. We have checked all the references to avoid any issues.
